# Clinical vitamin-A deficiency and associated factors among pregnant and lactating women in Northwest Ethiopia: a community-based cross-sectional study

**DOI:** 10.1186/s12884-019-2663-2

**Published:** 2019-12-18

**Authors:** Abebaw Baytekus, Amare Tariku, Ayal Debie

**Affiliations:** 1 Amhara National Regional State Health Bureau, Tikldengay Health Center, Tikldengay, Ethiopia; 2 Department of Human Nutrition, Institute of Public Health,College of Medicine and Health Sciences, University of Gondar, Gondar, Ethiopia; 3 Department of Health Systems and Policy, Institute of Public Health,College of Medicine and Health Sciences, University of Gondar, Gondar, Ethiopia

**Keywords:** Night blindness, Bitot’s spot, Pregnancy, Lactating, Vitamin A deficiency, Ethiopia

## Abstract

**Background:**

Vitamin A deficiency is known for its adverse health consequences, such as blindness, growth retardation and death. To curb the problem, Ethiopia has implemented various public health measures although little has been done to examine the deficiency among pregnant and lactating women. As a result, this study assessed the prevalence of Vitamin A deficiency and associated factors among pregnant and lactating women in Lay Armachiho district, northwest Ethiopia.

**Methods:**

A community-based cross-sectional study was conducted on pregnant and lactating women in Lay Arimachiho district, northwest Ethiopia, using the multistage systematic sampling technique to select participants. The binary logistic regression model was fitted to test the effect of exposure variables, and the Adjusted Odds Ratio (AOR) with a 95% Confidence Interval (CI) and *p*-value < 0.05 were computed to identify the significance and the strength of the associations of variables with Vitamin A deficiency.

**Results:**

The study revealed that 13.7% of the pregnant and lactating women had night blindness and 0.4% had also Bitot’s Spot. Over 35 years of age of mothers (AOR = 2.74; 95%CI: 1.15,7.43), less than USD 22.7 household monthly income (AOR = 8.9; 95%CI: 4.54,21.73), and poor hand washing practices after toilets (AOR = 8.87; 95% CI: 4.43,18.68) were positively associated with VAD, while mothers’ access to the media (AOR = 0.20; 95%CI:0.07, 0.59), formal education (AOR = 0.09; 95% CI: 0.03, 0.41), over 18 years of age at first marriage (AOR = 0.19; 95%CI: 0.08,0.36), and no fasting (AOR = 0.14; 95%CI: 0.04,0.46) were negatively associated.

**Conclusions:**

Maternal Vitamin A deficiency was the major public health problem in Lay Armachiho district. Over 35 years of age of mothers, less than USD 22.7 household monthly income and poor hand washing practices after toilets were high risks for VAD, while mothers’ access to the media, formal education, over 18 years at first marriage, and no fasting were low risks. Therefore, community awareness about the risk of early marriage, poor hand hygiene practices after toilets, and fasting during pregnancy and lactating period were essential. Organizations working on maternal health need to focus on mothers with low incomes in order to reduce their deficiency in Vitamin A.

## Background

Vitamin A Deficiency (VAD) is a major public health problem worldwide and a known cause of blindness, growth retardation and death, specially in developing countries [1]. It may also cause infertility,miscarriage, dry skin and hair as well as gastroenteritis, throat and chest infections [2–4]. The problem is common among children, pregnant and lactating mothers whose the requirement for vitamin-A is high because they need to compensate for their execessive physiological demand [5].

The cut-offs to declare the public health importance of clinical VAD are set to ease policy decisions. Accordingly, maternal VAD is considered as a public health problem if ≥5% or 0.5% of the mothers are affected with night blindness (XN) or Bitot’s Spot (X1B), respectively. According to this criteria, Vitamin-A deficiency is a worldwide public health problem. Hence, 7.8% of pregnant women are vitamin-A deficienct [6]. Furthermore, 9.8 million women worldwide are at risk for VAD. The highest burden of prenatal VAD was noted in African (9.8%) and South-East Asian (9.9%) [6, 7]. Low socio-economic status and impaired health conditions and dietary intake are commonly reported attributes of maternal VAD [8–14].

A few local studies reported that VAD had severe public health importance in both children and mothers [8, 9, 15–17]. The limited literature showed that 17.3 and 37.9% of pregnant mothers in north and south Ethiopia had night blindness and sub-clinical VAD (serum retinol < 0.7 μmol/L) [8, 9]. Considering the severity of the problem, high dose (200,000 IU) of vitamin-A has been universally provided for all lactating mothers. Yet, the micronutrient supplementation strategy did not target non-pregnant and non-lactating mothers to optimize the preconception maternal vitamin-A reserve [18]. Inspite of the fact that maternal breast milk vitamin-A concentration directly determines vitamin-A intake of breastfed children [9, 19–21], less research attention has been given to investigate the burden and the factors associated with maternal VAD among lactating women. Therefore, this study aimed to assess the prevalence of clinical VAD and associated factors among pregnant and lactating mothers in Lay Armachiho district, northwest Ethiopia.

## Methods

### Study settings and design

This community based cross-sectional study was conducted in Lay Armachiho district from February to March 2017. The district is situated in North Gondar administrative zone, the Amhara National Regional State, 210 km from Bahir Dar, capital of the region. According to the 2016/17 Central Stastical Agency estimation, the total population of the district was 140,417; pregnant and lactating mothers accounted for 3.36 and 3.09% of the population, respectively. Cereals, grains, roots and tubers are the commonest food products of the district.

### Population and sampling procedure

All pregnant and lactating women who lived in Lay Armachiho district were the source population, while all pregnant and lactating mothers in slected kebeles of the district were the study population. Lactating woman was defined as a breastfeeding mother who had less than one year old children.

The sample size was determined using the single population proportion formula considering a 95% level of confidence, 4% margin of error and a prevalence of maternal night blindness in Tahitay Koraro district, Tigray Region of 17.3% [9]. A 10% adjustment for non-response rate and 2 design effect yielded a sample of 754. Sample size for the second objective (determinants of VAD) was also calculated by assuming a 22% proportion of night blindness among pregnant and lactating mothers aged over 35 years, and less than ETB 500 household monthly income (24%) [9], 80% power, 95% level of confidence, 10% non-response rate and 2 design effect yielded 317 and 517, lower than the sample (754) for the first objective. Thus, 754 was taken as the final sample.

The multi-stage systematic sampling technique was used to select eligible participants; 6 of the 31 kebeles (lowest administration units) were selected by the lottery method. Then, 392 and 362 pregnant and lactating women were proportionally allocated to the selected kebeles. Finaly, the systematic sampling technique was employed to select eligible participants, and pregnancy was confirmed by mothers’ own reports.

### Measurments

Vitamin A deficiency was clinically confirmed by night blindness and Bitot’s Spot, while history of night blindness (dafint) was elicited by asking mothers in their local language for a word that stood for night blindness. Information on whether a woman faced any difficulty in identifying objects in dim light, especially at sun set, was collected [22]. On the other hand, mothers with opaque whitish/cheezy appearance deposits on sclera of their eye/s were deemed as having Bitot’s Spot [22, 23]. Consequently, if participants had at least one of the clinical signs (night blindness or Bitot’s Spot), the woman was defined as Vitamin-A deficient.

A standardized tool was used to measure the dietary diversity of the participants. The tool comprised 14 food groups, and food items consumed by participants in the previous 24-h were labeled as “food groups”. The final figures participants scored of the maximum of 14 based on their consumption of diversified food were categorized as “low”, “medium” and “high” if they reported to have consumed ≤3, 4–5 and ≥ 6 food groups, respectively [24]. Furthermore, a seven-day quasi-food frequency questionnaire was used to estimate mothers’ dietary intake for vitamin-A rich food. Participants were requested to report the number of days they ate the listed vitamin-A rich food groups one week before the data collection. In this study, pregnant or lactating mothers were also considered as fasting when they didn’t consume any thing for a minimum of nine hours (morning to 3:00 PM) except weekends, and couldn’t take any animal products at any time (day and night) for at least one month before the actual data collection.

### Data collection tools and procedures

A structured interviewer administered questionnaire was developed by reviewing literatures [8, 9, 24], and the questionnaire was first developed in English and translated into Amharic and back to English to maintain consistency. Six deploma level and two BSc degree graduate nurses were recruited to collect data and supervise the process, respectively. A two-day training was given to both groups on how to identify the clinical features of VAD (night blindness and/or Bitot’s Spot), interview techniques and data collection procedures. The questionnaire was modified based on the pre-test administered at Musie Bamb kebele on 38 mothers.

### Data management and analysis

Data were entered and analysed using Epi Info version 7 and SPSS version 20, respectively after cleaning to check accuracy, consistency and the identification of missed values. Descriptive statistics, such as frequency distributions, percentages, means, and standard deviations were used to summarize variables. A binary logistic regression model was fitted to test the effect of exposure variables on VAD. First, bivariable analysis was carried out to examine the effect of each independent variable on the outcome variable. Variables with *p*-values of < 0.2 in the bivariable analysis were fitted into the multivariable analysis. In the final model, independent variables with *p*-values of < 0.05 were considered as having statistically significant association with VAD. The strength of associations was determined using the adjusted odds ratio with 95%CI.

## Results

### Socio-economic and dietary habit related characteristics

A total of 742 pregnant and lactating women participated in the study with a response rate of 98.4%. The mean age of respondents was 30.4 ± (6.5 SD) years, while the mean age at first marriage was 16.5 ± (3.6 SD) years. Nearly all (98.8%) of the respondents were married. Subsistence farming (92.65%) was the major source of livelihood for the women in the study area (Table 1).

**Table 1:** Socio-demographic and economic characteristics of pregnant and lactating women, Lay- Armachiho district, northwest Ethiopia, 2017

Variables	Category	Frequency	Percent(%)
Maternal status	Pregnant	381	51.3
	Lactating	361	48.7
Maternal education	No formal education	477	64.3
	Formal education	265	35.7
Husband education	No formal education	393	53.0
	Formal education	349	47.0
Maternal occupation	House wife	703	94.7
	Government employee	39	5.3
Husband occupation	Farmer	687	92.6
	Government employee	55	7.4
Distance to source of water (minutes)	< 15	216	29.1
	15–30	298	40.2
	> 30	228	30.7
Diarrhea in the last 2 wks	Yes	711	95.8
	No	31	4.2
Wash hands with soap/ash after toilet	Not	147	70.6
	Sometimes	524	19.8
	Always	71	9.6
Age of mothers in years	< 25	136	18.3
	25–35	377	50.8
	> 35	229	30.9
Household monthly income (USD)	< 22.7	129	17.4
	22.7–45.5-	245	33.0
	> 45.5	368	49.6
Age at first marriage in years	< 18	473	63.7
	> 18	269	36.3
Treatment of water	Yes	552	74.4
	No	190	25.6

### Nutrition and obstetric related chacterstics

**Fig. 1:**
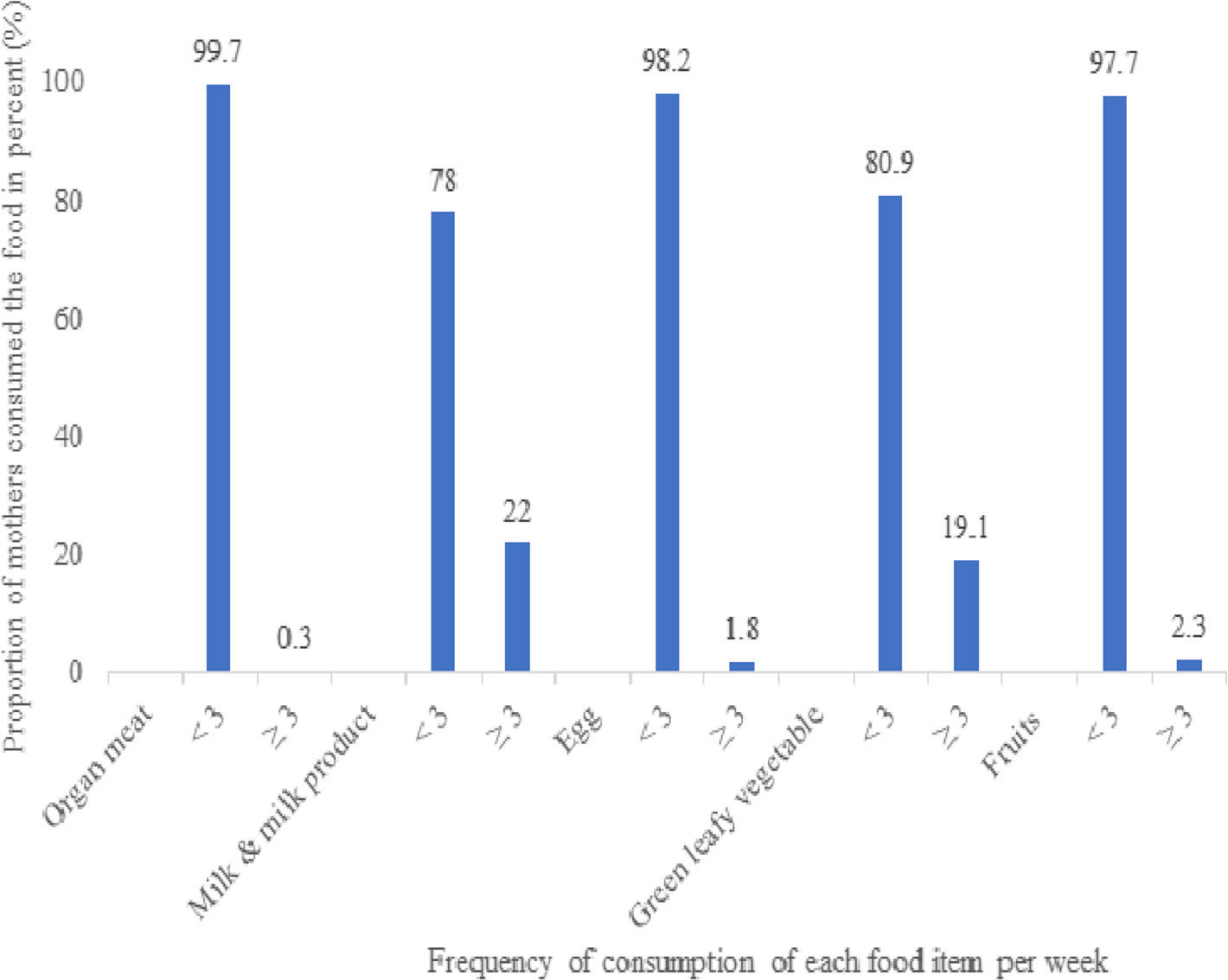
Vitamin-A rich food intake in the previous one week at time of data collection among pregnant and lactating women in Lay-Armachiho district, northwest Ethiopia, 2017

Nearly one-third (32.9%) of the respondents had two or more under-five children, and 88.0% had antenatal care follow-ups. The majority (81.2%) of the respondents were well-nourished (MUAC > 23 cm). More than one-third (41.0%) had low dietary diversity scores (Table 2). About 22 and 19.1% of the mothers consumed milk and green leafy vegetables three or more times per week, respectively (Fig. 1).

**Table 2:** Nutrition and obstetric related characteristics of pregnant and lactating women, Lay- Armachiho district, northwest Ethiopia, 2017

Characteristics(*N* = 742)	Category	Frequency	Percent(%)
Number of < 5 children	< 2	498	67.1
	> 2	143	19.3
Total number of births	< 5	412	55.5
	> 5	259	34.9
Birth spacing	< 3 years	165	22.2
	> 3 years	460	62.0
Mothers MUAC	< 23 cm	140	18.9
	> 23 cm	602	81.1
Fasting	Yes	571	77.0
	No	171	23.0
Families eat organ meat	Yes	503	67.8
	No	239	32.2
Meal frequency	> 3 meals	570	76.8
	3 meals	95	12.8
	< 2 meals	77	10.4
Dietary diversity score	Low	304	41.0
	Medium	355	47.8
	High	83	11.2

### Knowledge of vitamin-a related issues

About 40 % (41%) of the women had no information about VAD; over half (52.3%) did not know the signs and symptoms of VAD. More than two-thrids (71.8%) believed that VAD could not be prevented with diet (Table 3).

**Table 3:** Knowledge of pregnant and lactating women on Vitamin-A in Lay-Armachiho district, northwest Ethiopia, 2017

Characteristics	Category	Frequency	Percent (%)
Heard about VAD	Yes	438	59.0
	No	304	41.0
Mentioned sign of VAD	Weakness	36	4.9
	Becomes sick	27	3.6
	night blindness	176	23.7
	Total blindness	115	15.5
	Don’t know	388	52.3
Causes of VAD	Poor intake of variety of food	84	11.3
	Eating too little food	61	8.2
	Don’t know	597	80.5
Prevention of VAD with foods	Yes	209	28.2
	No	533	71.8
Prevention of VAD	Vitamin A rich food	96	12.9
	Vitamin A added food	2	0.3
	Vitamin A supplementation	35	4.7
	Don’t know	609	82.1
Mentioned vitamin A rich food sources	Green leafy vegetables	79	10.6
	Fruits	31	4.2
	Eggs	13	1.8
	Organ meat	4	0.5
	Milk and milk product	24	3.2

### Vitamin-A deficiency

Out of 742 the pregnant and lactating women, 13.7% had night blindness and 0.4% had also Bitot’s Spot. Of these, 6.6 and 7.1% were lactating and pregnant mothers, respectively.

### Factors associated with VAD

In the multivariable analysis, the odds of VAD among pregnant and lactating women who had formal education were 91% less likely (AOR = 0.09; 95% CI: 0.03, 0.41) compared to women who had no formal education. The finding indicated that the proportion of VAD among women who had no formal education was 20.6%, that is,19% higher compared with those who had formal education. Moreover, lesser odds of VAD were noted among women who had access to the media (AOR = 0.20; CI:0.07, 0.59) by 80%. Accordingly, only 3.72% of the participants had VAD among women with exposure to radio/TV, 13% lower compared with women with no access to the media. Women who had no fasting habits (AOR = 0.14; CI: 0.04,0.46) were also 86% less likely to have VAD. Similarly, the magnitude of VAD among fasting women was 16.43% and only 4.71% among non-fasting mothers. Women whose age at first marriage was above 18 years (AOR = 0.19; CI: 0.08,0.36) had lesser VAD compared with their counterparts. The study showed that 4.46 and 19.03% of pregnant and lactating women had VAD among participants whose age at first marriage was above and below 18 years, respectively. In contrast, higher odds of VAD were noted among women whose households’ monthly income was less than US$ 22.7 (AOR = 8.9; 95%CI: 4.54,21.73) compared with their counterparts. The study indicated that the prevalence of VAD among respondents who had less than US$ 22.7 monthly household income were 33.33 and 9.51% more affected than those whose monthly income was more than US$ 45.5. Participants who had no hand washing practices after toilets (AOR = 8.87; 95% CI: 4.43,18.68) were 8.87 times more likely to face VAD compared with women who washed hands after toilets. Women over 35 years of age (AOR = 2.74; CI: 1.15,7.43) were 2.74 times more likely to have VAD compared with those 25–35 years of age. This finding showed thatabout 20.09% of the women over 35 years and 9.56% of mothers below 25 years had VAD. Furthermore, this study indicated that 16.45% of women who had low dietary diversity faced VAD (Table 4).

**Table 4:** Factors associated with VAD among pregnant and lactating women in Lay- Armachiho district, northwest Ethiopia, 2017

Variables	Category	VAD		Proportion of VAD (%)	COR(95%CI)	AOR(95%CI)
		Yes	No			
Maternal education	No formal	98	379	20.55	1	1
	Formal	4	261	1.51	0.06 (0.02,0.16)	0.09 (0.03, 0.41)*
Listen radio/TV	Yes	7	181	3.72	0.58 (0.10,0.45)	0.20 (0.07,0.59)*
	No	95	459	17.15	1	1
Diarrhea in last 2 wks	Yes	9	22	29.03	2.72(1.22,6.80)	1.75 (0.37,4.51)
	No	93	618	13.08	1	1
Household monthly income (USD)	< 22.7	43	86	33.33	4.76 (2.87,7.89)	8.90 (4.54,21.73)*
	22.7–45.5	24	221	9.80	1.03 (0.59,1.79)	1.71 (0.79,4.54)
	> 45.5	35	333	9.51	1	1
Age at first marriage in years	< 18	90	383	19.03	1	1
	> 18	12	257	4.46	0.20 (0.11,0.37)	0.19 (0.08,0.36)*
Age of women in years	25–35	43	334	11.41	1	1
	< 25	13	123	9.56	0.82 (0.45,1.43)	0.87 (0.43,2.62)
	> 35	46	183	20.09	1.95 (1.26,3.22)	2.74 (1.15,7.43)*
Dietary diversity score	Low	50	254	16.45	1	1
	Medium	44	311	12.39	0.72 (0.46,1.11)	0.93(0.71,1.34)
	High	8	75	9.64	0.54 (0.25,1.19)	0.67 (0.43,1.51)
Wash hands with soap/ash after toilet	Sometimes	48	476	9.16	1	1
	No hand wash	52	95	35.37	5.43 (3.46,8.51)	8.87 (4.43,18.68)*
	Always	2	69	2.82	0.29 (0.07,1.21)	0.08 (0.02,0.76)*
Number of births	< 5	50	362	12.14	1	1
	> 5	52	278	15.76	1.35 (1.07,2.53)	1.21 (0.52,3.31)
No of < 5 children	< 2	62	436	12.45	1	1
	> 2	40	204	16.39	1.38 (0.99,2.69)	1.13 (0.48,5.12)
Fasting	Yes	94	478	16.43	1	1
	No	8	162	4.71	0.25 (0.12,0.52)	0.14 (0.04,0.46)*

*Significant at p-value < 0.05; AOR: Adjusted Odds Ratio; COR: Crude Odds Ratio

## Discussion

This study aimed to asses the prevalence of VAD and associated factors among pregnant and lactating mothers in Lay Armachiho district, northwest Ethiopia. The findings showed that 13.7% (95% CI, 11.3,16.3) of the pregnant and lactating women had night blindness and 0.4% had Bitot’s Spot. The reported burden of night blindness and Bitot’s Spot confirmed the public health importance of VAD. One of the main reasons for high VAD might be the low Vitamin A supplementation during pregnancy and postpartum period. In this study, Vitamin A supplementation among mothers was only 4.7%.

The prevalence of night blindness was consistent with that of a study conducted in the Republic of Congo (16%) [10] and Pakistan (16.2%) [25]. Neverthless, the finding was lower than those of studies conducted at Neader Adet (18.6%) [11], Tahitay Koraro (17.3%) [9], Sidama zone (37.9%) [8], Bangladish (37%) [26], and Nepal (21%) [27]. However, our result was higher than those of studies conducted in Atsede-Tsimbla (1.22%) [16], Wukro (5.8%) [15], Ethiopia (1.8%) [12], East Med (7.8%), West Pacific (5%) [28], Cameroon (6%), Ghana (7.7%), Congo (8%), Zambia (5.7%), Uganda (8.3%), and Tanzania (2.7%) [7, 29]. In this study, the prevalence of Bitot’s Spot (0.4%) was lower than the results of studies conducted in the pastoral areas of Ethiopia (1.6%), among grain farmers (1.1%), national level (1%) [12], and Congo (19%) [10]. The finding was consistent with that of cash-crop growers (0.4%) but higher than that of *enset* cultivating zones (0.0%) [12]. The possible explanation for the variations might be differences in study settings. That means, some studies were conducted in urban communities among well informed participants. Differences in study periods might also affect the Vitamin A status of mothers.

In this study, pregnant and lactating women over 35 years of age were 2.74 times more likely to be vitamin A deficient compared to mothers aged 25–35 years. This finding was supported by those of studies conducted in Tahtay Koraro of Tigray region [9], Sidama zone, southern Ethiopia [8] and rural Terai of Nepal [30], perhaps because mothers aged over 35 years had more pregnancies and births which depleted their vitamin A storage.

Households with less than USD 17.6 monthly income were 8.9 times more likely to be vitamin A deficient than those whose household monthly income was USD > 35.1. The result was similar to those of studies reported in Tahtay Koraro, Tigray region, and rural Cambodia [9, 13], perhaps because women who had relatively moderate income could get or consume more vitamin A rich foods than the poorest; the richest might also use maternal health services more than the poorest.

Women who had formal education and access to the media programs were 91 and 80% less likely to be vitamin A deficient compared to their counterparts, respectively. This finding was consistent with those of studies done in Sidama zone, southern Ethiopia [8], Bangladish [26], and rural south India [14]. The possible explanation might be that educated and mass media program accessing women had better information and knowledge about the sources of vitamin A rich foods and prevention strategies for vitamin A deficiency.

Women with no fasting were 86% less likely to be vitamin A deficient than those who were fasting, perhaps because women who fasted had poor vitamin A intake, negatively affecting their hepatic store. Poor dietry intake coupled with depleted liver storage of Vitamin-A predisposed mothers to VAD.

Women who always washed their hands after toilets with ash or soap were less likely to be vitamin A deficient by 92% than women who only sometimes did that. The finding was supported by those of studies in Bangladish [26] and Tahtay Koraro [9]. The possible explanation might be that women who had poor hand washing practices after toilets were highly prone to infections due to contaminations that subsequently led to disease-induced VAD. Furthermore, this study indicated that a high proportion of women who had low dietary diversity scores might have enhanced their exposure to vitamin A deficiency. This result was supported by a finding in South Africa [31]. This might be due to a low intake of vitamin A enrich foods as a result of low diversification of foods. The limitation of this study was the use of maternal self-report of amenorrhea, increasing uterine size to detect pregnancy for mothers who had no pregnancies test results. This might have reduced the reliability of pregnancies since the causes for amenorrhea might have been other conditions, for instance, menustrial/ endocrine disorders, malnutrition etc. Another limitation was the reliance on symptoms and signs of vitamin A without measuring the serum retinol level. Seasonal variations of maternal Vitamin A status were not addressed in this study. As it employed the cross-sectional study design, over work was bound to miss/ lack the comparative longitudinal characterstics. Besides, it was not able to describe other possible causes of the night blindness.

## Conclusion

Overall, maternal VAD was a major public health problem in the study area. Over 35 years of age, age at first marriage > 18 years, no fasting, poor hand washing practices after toilets, and less than USD 22.7 household monthly income were the factors associated with maternal VAD. Therefore, strengthening the awareness of pregnant and lactating women about the risk of early marriage, encouraging hand washing practices after toilets, and no fasting during pregnancy and lactating periods are recommended. Mothers had better access to the media, particularly health related programs. Women should also take a more diversified food to get more Vitamin A since vitamin A supplementation noted in this study was very low, healthcare providers need to improve vitamin A supplementation to maintain women’s Vitamin A stasus because pregnancy and lactating periods require more vitamin A. Furthermore, researchers have to focus on measuring retinol concentration and assess seasonal variations of maternal VAD using comparative longitudinal designs in order to examine the Vitamin A status of exposed and unexposed groups of mothers. Organizations working on maternal health need to focus on low income mothers to reduce their VAD related problems.

## Supplementary information

**Additional file 1.** : English version questionnaire.

## Data Availability

Data will be available upon reasonable request from the corresponding author. However, the data cannot be made public to maintain mother’s privacy and legal reasons as it contains private health information along with identifiers.

## Abbreviations

ARI: Acute Respiratory Infection
MMR: Maternal Mortality Ratio
SPSS: Statistical Package for Social Sciences
UNICEF: United Nation International Children Fund
VAD: Vitamin A Deficiency
VADD: Vitamin A Deficiency Disorder
WHO: World Health Organization
X1B: Bitot’s Spot
XN: Night Blindness
