# Supplementary material for: Clinical vitamin-A deficiency and associated factors among pregnant and lactating women in Northwest Ethiopia: a community-based cross-sectional study
Source: BMC Pregnancy Childbirth. 2019 Dec 18;19:506. doi: 10.1186/s12884-019-2663-2 (PMC6921426; doi:10.1186/s12884-019-2663-2)
Supplement: Supplementary file 1 — Additional file 1. : English version questionnaire. [file 12884_2019_2663_MOESM1_ESM.docx]

**English version questionnaire to assess VAD among pregnant & lactating women in Lay-Armachiho district**

| **Part I: Socio- demographic and economic characteristics of respondents** | | |  |
| --- | --- | --- | --- |
| No | Question | Possible responses | Remark |
| 101 | Age of mothers | -------------- in years |  |
| 102 | Religion | 1. Orthodox  2. Muslim  3.Catholic  4.Protestant  5. Others, specify--------- |  |
| 103 | Marital status | 1. Single  2.Married  3.Separated  4.Divorced  5.Widowed |  |
| 104 | What was your age at the time of your first marriage? | **---------------**in years |  |
| 105 | Maternal Educational status | 1. Unable to read and write  2. Able to read and write  3. Elementary school(1-8)  4. High school(9- 12)  5. Above high school |  |
| 106 | Husband Educational status | 1. Unable to read and write  2. Able to read and write  3. Elementary school(1-8)  4. High school(9-12)  5. Above high school |  |
| 107 | Husband occupation | 1. Farmer  2. merchant/salesman  3. Government employee  4. Others, specify-------- |  |
| 108 | Mother’s occupation | 1. Housewife.  2.Farmer  3. Merchant/salesman  4. Government employee  5. Others, specify-------- |  |
| 109 | What is the the average household monthly income in ETB? | ----------------- ETB |  |
| 110 | Do you have television and radio? If her answer is No go to question 112. | 1.Yes  2.No |  |
| 111 | How often have you listened television and/or radio? | 1. None 2. Sometimes 3. Always |  |
| 112 | Distance of your house to water source (minutes) | 1. < 15  2. 15-30  3. > 30 |  |
| 113 | Does your source of water is treated? | 1.Yes  2.No |  |
| 114 | How often have you washed your hand with soap/ash after toilet? | 1.No hand wash  2. Sometimes  3. Always |  |
| 115 | Did you have diarrhea within the last 2weeks? | 1. Yes 2. No |  |

| **Part II: Mother’s status of night blindness and Bitot’s spot** | | | |
| --- | --- | --- | --- |
| No | Question | Possible response | Remark |
| 201 | Do you have difficulty of seeing with decreased light or at night or “Dafint” (a local term for night blindness)? | 1.Yes  2.No |  |
| 202 | Is there Bitot’s Spot in either eye of a woman? (see) | 1.Yes  2.No |  |

| **Part III : Maternal , pregnancy & diet characteristics of respondents** | | | Remark |
| --- | --- | --- | --- |
| No | Question | Possible responses |  |
| 301 | Women’s current status | 1. Pregnant 2. Lactating |  |
| 302 | How many under five years children do you have in the household? | ---------------- |  |
| 303 | How many children born alive? | --------------- |  |
| 304 | How many total births did you encounter? | --------------- |  |
| 305 | What is your regular birth spacing? (in years) | --------------- |  |
| 306 | MUAC of woman | ------------- cm |  |
| 307 | Have you been fasting? | 1.Yes  2. No |  |
| 308 | If yes for Q307, for how long? (Fill each of the spaces) | 1. Hours per day------ 2. Days/month-------- 3. Consecutive months------------- 4. If any food restriction or other issue (specify) --------- |  |
| 309 | Have you taken vitamin A supplementation (For lactating women only)? | 1. Yes 2. No |  |
| 310 | How often have you eaten the following food items per week? | 1. Organ meat (liver, heart, kidney) ……. 2. Egg……… 3. Milk and milk product……. 4. Green leafy vegetables   Cabbage……………  Carrot……………….  Green pepper……….  Spinach…………….  Pumpkin……………  Kale………………  Lettuce……………   1. Fruits   Orange……………  Papaya………………..  Avocado………………  Mango…………………….  Banana…………………… |  |

| **Part IV: Maternal knowledge about night blindness** | | |  |
| --- | --- | --- | --- |
| 401 | Have you heard about vitamin A deficiency or lack of vitamin A? | A. Yes  B. No  C. Don’t know |  |
| 402 | *If Yes:*  Can you tell me how you can recognize someone who lacks vitamin A in his or her body? | 1. Weakness/feels less energetic 2. Be more likely to become sick (less immunity to infections) 3. Eye problems: night blindness (inability to see at dusk and in dim light), dry 4. eyes, corneal damage, blindness 5. Others 6. Don’t know |  |
| 403 | What are the causes of lack of vitamin A in the body? | A. Poor variety of foods  B. Eat too little food/ not eat much (poor intake)  C. Others------------  D. Don’t know |  |
| 404 | How lack of vitamin A in the body can be prevented? | A. Eat/feed vitamin-A-rich foods-having/ giving a diet rich in vitamin A  B. Eat/feed foods fortified with vitamin  C. Give vitamin A supplements/sprinkles  D. Others ------------- |  |
| 405 | Do you know any animal-source of foods that are rich in vitamin A? | 1. Liver 2. Kidney 3. Heart 4. Egg yolks 5. Milk, cheese, yogurt or other dairy product 6. Others-------------- |  |
| 406 | Do you know any green and orange-colored vegetables that are rich in vitamin A? | 1. Cabbage 2. Carrot 3. Pumpkin 4. Green pepper 5. Spinach 6. Kale 7. Others-------- |  |
| 407 | Do you know any fruits (orange- or yellow-colored non-citrus fruits) that are rich in vitamin A? | 1. Mango 2. Papaya 3. Avocado 4. Orange 5. Others --------- |  |

| **Part V: Dietary diversity score measuring questions to assess women’s feeding for the last 24 hours** | | | |
| --- | --- | --- | --- |
| Question number | Food group/s | Examples | **Remark** |
| 501 | Cereals | corn/maize, teff, rice, wheat, sorghum, millet or any other grains or foods made from these (e.g. bread, noodles, porridge or other grain products) e.g. enjera, kita, kolo, nifro | 1. Yes 2. No |
| 502 | Vitamin A richvegetables andtubers | pumpkin, carrots, squash, or sweet potatoes thatare orange inside + *other locally available vitamin-A rich vegetables (e.g. red pepper)* | 1. Yes  2. No |
| 503 | White tubers and roots | white potatoes, false banana (enset), white yams, white cassava, or other foods made from roots | 1 . Yes  2. No |
| 504 | Dark green leafyvegetables | dark green/leafy vegetables, including wild ones +*locally available vitamin-A rich leaves such asamaranth, , kale, spinach, pumpkin leaves, etc.* | 1.Yes  2. No |
| 505 | Other vegetables | other vegetables (e.g. tomato, onion, eggplant), including wild vegetables | 1. Yes 2. No |
| 506 | Vitamin A richfruits | ripe mangoes, cantaloupe, apricots (fresh or dried),ripe papaya, dried peaches + *other locally availablevitamin A-rich fruits* | 1. Yes 2. No |
| 507 | Other fruits | other fruits, including wild fruits | 1. Yes 2. No |
| 508 | Organ meat (iron-rich) | liver, kidney, heart or other organ meats or blood-based foods | 1. Yes 2. No |
| 509 | Fresh meats | beef, pork, lamb, goat, chicken, or other birds | 1. Yes 2. No |
| 510 | Eggs | chicken, duck, guinea hen or any other egg | 1. Yes 2. No |
| 511 | Fish | fresh or dried fish | 1. Yes 2. No |
| 512 | Legumes, nuts and seeds | beans, peas, lentils, chickpea, nuts, seeds or foods made from these | 1. Yes 2. No |
| 513 | Milk and milk products | milk, cheese, yogurt or other milk products | 1. Yes 2. No |
| 514 | Oils and fats | oil, fats or butter added to food or used for cooking | 1. Yes 2. No |
